# Survey of Programs Used to Detect Alternative Splicing Isoforms from Deep Sequencing Data *In Silico*

**DOI:** 10.1155/2015/831352

**Published:** 2015-09-03

**Authors:** Feng Min, Sumei Wang, Li Zhang

**Affiliations:** Department of Infectious Diseases, The Affiliated Chenggong Hospital of Xiamen University, The 174th Hospital of the Chinese People's Liberation Army, Xiamen, Fujian 361000, China

## Abstract

Next-generation sequencing techniques have been rapidly emerging. However, the massive sequencing reads hide a great deal of unknown important information. Advances have enabled researchers to discover alternative splicing (AS) sites and isoforms using computational approaches instead of molecular experiments. Given the importance of AS for gene expression and protein diversity in eukaryotes, detecting alternative splicing and isoforms represents a hot topic in systems biology and epigenetics research. The computational methods applied to AS prediction have improved since the emergence of next-generation sequencing. In this study, we introduce state-of-the-art research on AS and then compare the research methods and software tools available for AS based on next-generation sequencing reads. Finally, we discuss the prospects of computational methods related to AS.

## 1. Introduction

Alternative splicing (AS) refers to the production of pre-mRNA via gene transcription to generate a number of mature mRNAs based on different splice modes, thereby increasing protein diversity. Since alternative splicing was discovered, studies have identified a large number of AS events in the human gene transcription process [1]. Based on high-throughput deep sequencing data, AS occurs in approximately 95% of the human genome [2]. AS is an important regulatory mechanism involved in the regulation of eukaryotic gene expression and proteome diversity [3]. The process is closely linked with many diseases, including cancer and diseases of the nervous system [4–6]. Thus, scholars in medicine, genetics, bioinformatics, and other fields have directed considerable research interest towards AS with the aim of identifying additional splicing events that could facilitate a deeper understanding of the AS regulatory mechanism.

Splice site recognition represents a key step in selective splicing research. Splice sites are used to predict the positions of exon/intron structures and splice site features, and splice site recognition is the traditional strategy used to predict alternative splice sites. Many algorithms, software, and databases for sequence alignment have emerged due to the application of first-generation sequencing. The research resources designed specifically for AS have gradually become richer, including a common ASD AS database [7]. However, the cost of first-generation sequencing is high; considerable efforts have been directed towards the goal of creating thousand- and hundred-dollar genome sequencing technology in the postgenomic era. Thus, the high throughput and low cost of next-generation sequencing technologies have provided a new stage for scientific research [8, 9].

AS was discovered in 1977 [10]. Subsequently, researchers realized the importance of AS due to its ability to regulate gene expression and facilitate protein diversity [11, 12]. The advantages of next-generation sequencing technology have opened a new stage of sequencing, and the study of the massive amounts of data generated by RNA-seq technology has become an important research direction.

RNA-seq (high-throughput RNA sequencing) represents a new method for the analysis of gene expression and transcriptomes. Many software tools and databases have appeared with the capacity to generate short sequence alignments and predictions on the basis of the alternative splice sites identified using RNA-seq.

In this study, we outlined the methods, software tools, and databases available for AS research under two-generation sequencing technologies. The effect of these factors on AS research was analyzed. Using RNA-seq data produced by the Illumina/Solexa sequencing platform as an example, we compared three common splice site prediction programs (HMMSplicer [11], SOAPsplice, and TopHat [8]) under conditions of different depths and sequence read lengths. The performance of each type of software was evaluated under different conditions by comparing the number of accurately predicted sites, the accuracy rate, and the error rate. Finally, we discussed the problems and challenges associated with using deep sequencing data to study AS.

## 2. Discovering Alternative Splicing Sites from Long DNA Sequences

**Figure 1:**
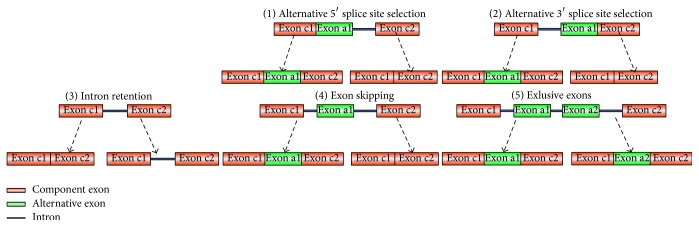
Five types of alternative splicing.

In addition to experimental methods, researchers predict potential AS events through the comparison between EST expression sequence tags and gene sequences. A large number of analyses and studies have validated the significance of the 3′ terminal splice acceptor site and 5′ terminal splice donor site in splicing events.  Figure 1 summarizes the five AS forms.

The study by Fairbrother et al. [13] on exons in the human genome revealed that the splicing enhancers ESE and ESS serve an important regulatory function in selective splicing. Black [14] demonstrated that the splicing enhancer ISE and silencer ISS are also important for the selection of splicing sites and recognition of exons and introns. Thus, the AS process in eukaryotic genes is determined not only by a splicing factor but also by a complex regulatory process.

The means of selective splicing mainly include the following.

1. Comparison analysis based on ESTs, mRNA, and gene fragments: EST comparative analysis was one of the earliest AS research methods. This method can identify certain AS events. However, EST has its own limitations, such as incomplete data, influence from genetic pollution, sensitive 3′ terminal, and high cost [15, 16]. Common comparison software programs include BLAT [17], Clustal [18], SIM4 [19], Ecgene [20], ASPIC [21], Spidey [22], GeneSeqer [23], and GMAP [24].
2. Using gene chip high-throughput technology: gene chip technology has facilitated the research upsurge in the whole gene transcriptome. A large number of AS events have been identified using this technology. Johnson et al. [1, 25] discovered many exon-skipping events by analyzing microarray data. However, the disadvantage of this method is that probe density is limited, and designing a probe based on the known sequence and data analysis is difficult.
3. Using machine learning methods for theoretical prediction: machine learning techniques have been widely used in various tasks in the field of bioinformatics, such as protein remote homology detection [26–29], microRNA identification [30, 31], protein binding site prediction [32], domain boundary identification [33, 34], DNA-binding protein prediction [35–37], protein structure prediction [38], enzyme classification [39, 40], gene regulation network construction [41], heat shock protein classification [42, 43], replication origin prediction [44, 45], nucleosome positioning sequence identification [46–48], CpG island methylation status prediction [49], translation initiation site prediction [50], promoter prediction [51], and microarray clustering [52, 53]. These machine learning based methods have achieved promising predictive performances. Therefore, some researchers have also applied common machine learning methods for theoretical predictions, such as support vector machine (SVM) [54, 55], weight matrices, the hidden Markov model, the quadratic discriminant function [56], and the neural network model [57]. The programs used for predicting splice sites based on these algorithms include HMMgene [58], NetGene2 [59, 60], geneID [61], GeneSplicer [62], and SpliceMachine [63].

## 3. Discovering Alternative Splicing Sites from Short Reads

The next-generation high-throughput sequencing technology developed rapidly after its emergence, thus enabling sequencing technology to move a step closer towards the thousand-dollar genome project. RNA-seq represents a new approach for gene expression and transcriptome studies. Currently, traditional AS research methods coexist with the development of the next-generation research methods. An increasing number of studies have been devoted to the development of new algorithms. In summary, next-generation high-throughput sequencing technology can provide a broad platform for AS due to its high efficiency and inexpensiveness.

However, RNA-seq also has shortcomings. The main challenge stems from read length. The read length of first-generation sequencing (i.e., Sanger sequencing) reaches approximately 1000 bp. The initial read length of RNA-seq was only approximately 25 bp. The read length is still relatively short, despite reaching 100 bp using Illumina/Solexa double-end sequencing [64].

### 3.1. Data Preprocessing

The first step in predicting an alternative splice site is to position the read on the reference transcriptome using RNA-seq data. However, the general analysis tools often position the reads on the reference genome because the transcriptome itself is not complete [8]. Short RNA-seq read lengths and incomplete transcriptomes cause the accuracy of this step to directly influence the accuracy of the prediction.

*Some data found in read mapping* can cross two exon-exon junctions [65]. This “read in junction” cannot be directly positioned on the genome sequence. This finding represents the key to studying alternative splice sites and identifying the critical region for exploring undetected splice events. Therefore, the processing strategy used to splice the read in junction is the key to predicting splice sites [66]. One approach for the treatment of read in junction is to position the reads onto the reference genome according to the currently known annotation of the exons. ERANGE [67] uses this method. Obviously, identifying new splice events is difficult using this approach. Another approach is to completely position the reads on the reference genome so that they can be divided into several different clusters. Reads with overlapping areas are classified into the same cluster. An exon region is delimited in each cluster [65]. Finally, the reads in junctions are positioned on the possible junctions. New splice events can be identified because the reads are based on known exon annotations. The splice site prediction software TopHat [8] uses this strategy.

Numerous software programs are specifically designed for the read mapping of RNA-seq data. These programs adopt the following algorithms: (1) the Smith-Waterman algorithm, such as BFAST [68] and SHRiMP [69]; (2) the two-way Burrows-Wheeler transform (BWT) algorithm, such as SOAPAligner [70]; (3) the BWT algorithm, such as Bowtie [71] and BWA [72]; and (4) the spaced-seed vacancy seed algorithm, such as MAQ [73]. Data compatibility should also be considered along with the choice of software. The formats of RNA-seq data generated by various sequencing platforms are different [74]. Thus, software versatility is affected by the styles and variety of formats it supports. Bowtie and BWA are relatively efficient, whereas SOAPAligner, BFAST, and MAQ have good tolerance for mismatches.

In addition to read mapping, we identified special software devoted to read assembly (i.e., de novo assembly). Few methods to study AS based on read assembly exist. However, read assembly has special roles in other biological information sciences. The typical read assembly software includes SHARCGS [75], SSAKE [76], and ALLPATHS [77]. The former two are assembled only for single sequence data, while the latter can be assembled for a pair of sequences from double-end sequencing. MAQ also has the ability to perform read assembly. Finally, sequence read archive (SRA) files are specialized for the storage of databases related to RNA-seq data for NCBI for inclusion into an AS database.

### 3.2. Alternative Splicing Prediction

The common AS site prediction software includes ERANGE, QPALMA [78], TopHat, MapSplice [79], SpliceMap, SOAPsplice, SplitSeek [80], and HMMSplicer. Current studies using RNA-seq to identify AS sites focus on locating splice sites, discovering new splice sites located as distantly as possible, and conducting next-step AS studies. Therefore, the accuracy and efficiency of predictions are key factors for the prediction software. Moreover, accuracy should be improved in order to predict more splice sites, while the error probability should be reduced; these factors differ for selected algorithms.

ERANGE was the earliest available method. It was the first program to use the read mapping method. In this method, the read is positioned on the reference genome based on known exon annotations. Thus, this method cannot be used to identify a new splice site. QPALMA adopts the machine learning strategy and trains support vector machines for site identification using known splice sites. Vmatch has been adopted for positioning. However, because the efficiency of Vmatch is not high enough compared with Bowtie, Vmatch is not used for comparing reads. TopHat first positions the sequence on the reference genome using Bowtie. MAQ successfully positions the sequence assembly on the reference genome. Then, a possible splice site is recognized based on the adjacent exons. Additionally, the sequences not positioned on the reference genome are collected to establish the vacancy seed index. Finally, the vacancy expansion is compared in order to obtain the possible splice sites. According to a test reported by the authors, TopHat processed 2.2 million reads per hour, whereas QPALMA processed approximately 180,000. However, the performance will be poor when the depth of sequencing is low or the intron is very short because the algorithm adopts exon islands.

SpliceMap consists of four main steps: half-read mapping, seeding selection, site search, and paired-end filtering. First, SpliceMap splits the read into halves. Alignment positioning is performed between each portion and the gene sequence. Then, the remaining half is positioned on the downstream region within the range of the longest intron. This approach requires the read length to be at least 50 bp. Therefore, SpliceMap cannot process read lengths <50 bp. When we compared SpliceMap with ERANGE, ERANGE discovered 160,899 sites, whereas SpliceMap accurately predicted 127,043 sites. Moreover, 24,274 of the 151,317 sites discovered by SpliceMap were not discovered by ERANGE, of which 23,020 represent new splice sites. However, these new sites are unconfirmed. The MapSplice software appeared after TopHat and SpliceMap. MapSplice is not based on the characteristics of splice sites or the length of an intron. It also has the potential to discover new sites and can adapt the length of the read.

The emergence of SOAPsplice improved the evaluation standard of splice site prediction software. SOAPsplice not only depends on the number of recognition splice sites but also emphasizes a high accuracy and low error rate. The experiment described in the next section revealed that the performance of SOAPsplice was comparatively outstanding. SplitSeek is strict with regard to the format of the input data and only supports data generated by ABI SOLiD. Moreover, because the input data are processed by a complete ABI transcriptome analysis tool, the application is not very wide. HMMSplicer is similar to SpliceMap but possesses several innovations. First, it divides the read into halves and compares halves with the genome sequence. The exon boundary (i.e., the 5′ terminal) is obtained using the hidden Markov model (HMM). Second, the remaining half is positioned downstream the first half to determine the boundary 3′ terminal of the intron. Both common (GT-AG, GC-AG, and AT-AC) and uncommon splice sites are recorded during this process. Finally, the scores of candidate loci are graded using the scoring algorithm.

### 3.3. Aligning Spliced Reads to the Reference Genome

Read lengths generated by all types of sequencing platforms are growing concomitant with the development of deep sequencing and RNA-seq technology. In the early days, read lengths were usually approximately 32 bp, and most of the software programs did not consider the location of the spliced reads on the reference genome. However, with the generation of longer reads, new requirements were put forward for locating software.

Reads mapping and alternative splicing detection are two steps in an analysis workflow. RNA read alignment is the precursor step and splice isoform detection is the successor step. Splice isoform detection tools include Cufflinks [81] and Scripture [82]. Cufflinks is a software tool for detecting the specific expression genes. If users have two groups of RNA-Seq data, such as ill and normal persons, it would be better to employ Cufflinks for the key genes detection. Scripture is a method for transcriptome reconstruction that relies solely on RNA-Seq reads and an assembled genome to build a transcriptome* ab initio*.

Researchers applied the preprepared splice site database when they began trying to align spliced reads to the reference genome. However, the existing annotation of the transcriptome was far from being perfect. Therefore, some researchers once again began using BLAT to locate reads.

The TopHat software program solved these problems and thus became widely used by researchers; moreover, its vision has been expanding in every release from its initial release. In addition to its ability to align spliced reads to the reference genome, TopHat can also predict possible splice sites. These splice sites play an important role in improving the annotation of the transcriptome. The initial vision of TopHat had many limitations; however, the adoption of new methods in the software updates has improved TopHat's performance.

With the development of sequencing technologies, reads with lengths >100 bp have been produced on a large scale. These reads may span one or more spliced sites, which introduces difficulty in aligning spliced reads. The SpliceMap software is capable of processing longer reads (read lengths > 50 bp). To process these long reads, SpliceMap divides the reads into overlapping short read fragments. Then, they are annotated with the locating information of whole reads based on the locating information of the short read fragments.

MapSplice is another package that aligns spliced reads to the reference genome, although it applies a different method. The MapSplice algorithm is suitable for all types of read lengths. It is similar to SpliceMap in that it does not use continuous aligning of the reads to create an exon library in advance. Because the MapSplice package does not depend on spliced read signal information when aligning reads, it can locate some reads that SpliceMap cannot align. It can also be used to predict new spliced reads with no spliced read signal information. Another advantage of the MapSplice package is its high efficiency compared with most other software.

Package SeqSaw was proposed by Wang et al. [83] and is totally different from TopHat and MapSplice. It was similar to the SpliceMap package in its early releases. However, SeqSaw use has dynamically changed to Hash Table to reduce the search space. The core algorithm of SeqSaw is focused on locating short reads to the genome. There are very few introns >400 Kb in the known mammalian genome. Thus, we can define intron lengths as being less than a certain value, with a default value of 400 Kb. Users can adjust the value according to the needs of different species or datasets. However, SeqSaw uses certain means and performs a large amount of optimization, which greatly reduces the search space.

**Figure 2:**
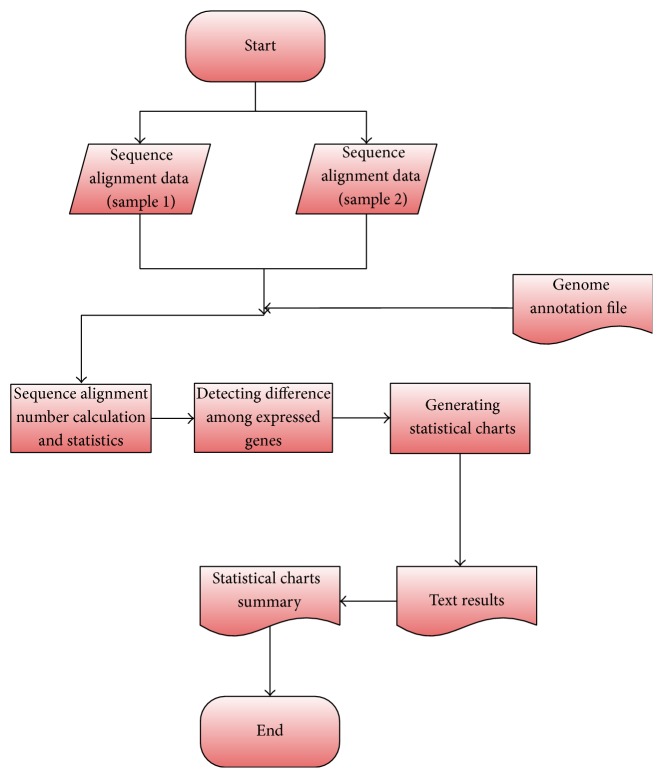
Working process of DEGseq.

The R package DEGseq [83] has been proposed to detect small changes in the genetic expression of each sample. It is used to assess the trend of background noise in MA due to technological repeats.  Figure 2 shows the working process of DEGseq.

The difference between a DNA aligner and an RNA aligner is that an RNA aligner can tolerate extra-long deletions (introns) while DNA aligners cannot [84]. Moreover, many RNA aligners are constructed based on DNA aligners (i.e., TopHat is built based on Bowtie). STAR is the latest and most popular RNA-seq alignment tools. In addition to unbiased de novo detection of canonical junctions, STAR can discover noncanonical splices and chimeric (fusion) transcripts and is also capable of mapping full-length RNA sequences [85].

## 4. Experiments Using State-of-the-Art Software Tools

HMMSplicer, SOAPsplice, TopHat, and STAR were used to perform the following analysis of Illumina/Solexa output data. The reference genome data are from the tenth human chromosome. The gene sequence was processed into RNA-seq sequences with different read lengths and different sequencing depths as the test data for SOAPsplice and TopHat. HMMSplicer does not support double-end sequencing data, so each pair of FASTQ data was merged into a FASTQ file as the test data for HMMSplicer.

**Figure 3:**
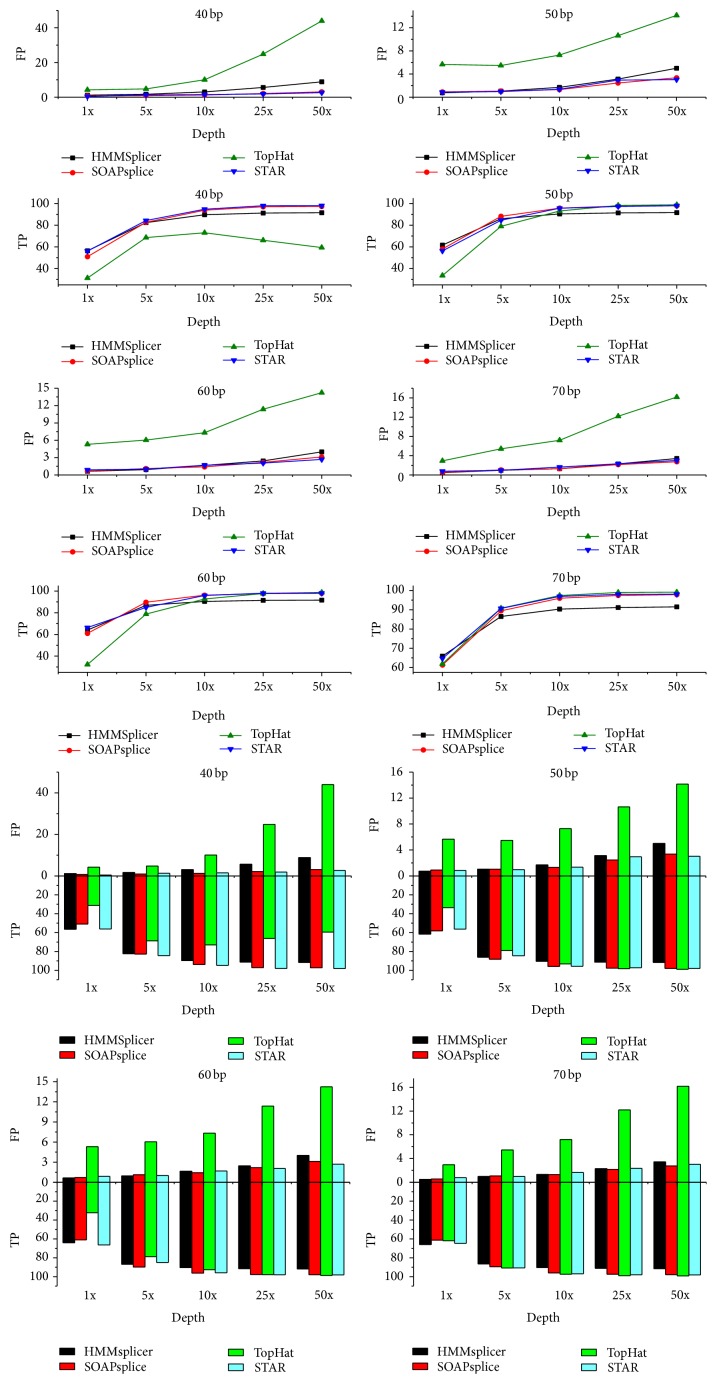
Comparison of HMMSplicer, SOAPsplice, STAR, and TopHat.

Figure 3 shows that, in the premise of the 50 bp read length, each type of software predicts an increase in the number of loci that increases with the development of sequencing technologies. The accuracy of TopHat is poorer compared with the other two programs within a sequencing depth range of 1x to 10x, and the error rate is still high. The accuracy of TopHat increased rapidly after the sequencing was deepened. SOAPsplice and TopHat performed well in the aspect of accuracy, although the error rate was significantly worse for TopHat. STAR works best among the four tested tools. SOAPsplice and STAR performed well in both aspects.

## 5. Conclusion

In this study, we analyzed and compared the current AS-associated algorithms and software. We summarized the present situation of AS. The read mapping, including AS and site recognition algorithms, remained the focus of the current research. We aimed to improve the algorithm's quality in order to increase the number of prediction sites as much as possible and to meet the high-accuracy rate. RNA-seq data size is very large due to the continuous development of next-generation sequencing technology. This study represents a broad platform for AS and other fields of bioinformatics. This review of experimental and research methods for AS may be helpful for other researchers.

Although high-throughput sequencing has given rise to an unprecedented opportunity for the study of AS, few scholars study AS based on RNA-seq data. Therefore, the available algorithms and software are not rich compared with those based on EST/cDNA theory. Significant differences are found in the alignment step between the algorithms and the software using next-generation technology. This step represents the critical step based on the study of RNA-seq data. The software tools and algorithms need to be considered in parallel as the read data becomes more massive [86]. Genome-wide analysis will be the hot topic for all alternative and epigenetic research fields [87]. Moreover, many of the special databases based on RNA-seq data are not perfect. The corresponding new research methods and databases will be perfected with the constantly developing study of AS.
